# Replicative aging in yeast involves dynamic intron retention patterns associated with mRNA processing/export and protein ubiquitination

**DOI:** 10.15698/mic2024.02.816

**Published:** 2024-02-23

**Authors:** Jesús Gómez-Montalvo, Alvaro de Obeso Fernández del Valle, Luis Fernando De la Cruz Gutiérrez, Jose Mario Gonzalez-Meljem, Christian Quintus Scheckhuber

**Affiliations:** 1 Tecnologico de Monterrey, Escuela de Ingeniería y Ciencias, Ave. Eugenio Garza Sada 2501, Monterrey, N.L., México.

**Keywords:** Saccharomyces cerevisiae, intron retention, replicative aging, mRNA processing, mRNA export, ubiquitination, transcription regulation

## Abstract

*Saccharomyces cerevisiae* (baker's yeast) has yielded relevant insights into some of the basic mechanisms of organismal aging. Among these are genomic instability, oxidative stress, caloric restriction and mitochondrial dysfunction. Several genes are known to have an impact on the aging process, with corresponding mutants exhibiting short- or long-lived phenotypes. Research dedicated to unraveling the underlying cellular mechanisms can support the identification of conserved mechanisms of aging in other species. One of the hitherto less studied fields in yeast aging is how the organism regulates its gene expression at the transcriptional level. To our knowledge, we present the first investigation into alternative splicing, particularly intron retention, during replicative aging of *S. cerevisiae*. This was achieved by utilizing the IRFinder algorithm on a previously published RNA-seq data set by Janssens *et al.* (2015). In the present work, 44 differentially retained introns in 43 genes were identified during replicative aging. We found that genes with altered intron retention do not display significant changes in overall transcript levels. It was possible to functionally assign distinct groups of these genes to the cellular processes of mRNA processing and export (e.g., *YRA1*) in early and middle-aged yeast, and protein ubiquitination (e.g., *UBC5*) in older cells. In summary, our work uncovers a previously unexplored layer of the transcriptional program of yeast aging and, more generally, expands the knowledge on the occurrence of alternative splicing in baker's yeast.

## INTRODUCTION

Organismal aging is a universal phenomenon characterized by a progressive and irreversible decline in physiological functions, accompanied by increased morbidity and mortality [1]. Extensive research across various model organisms and systems has aimed to decipher the underlying processes of aging [2]. This intricate phenomenon is governed by a complex network of molecular pathways that can exhibit both species-specific traits and conserved mechanisms [3]. Understanding the fundamental mechanisms that drive aging holds paramount importance, offering insights into the aging process across different systems [4].

The unicellular ascomycete *Saccharomyces cerevisiae* has emerged as a valuable model organism for investigating the mechanistic basis of aging [5]. Replicative aging in *S. cerevisiae* is defined by the limited number of daughter cells a mother cell can produce through the budding process [6]. It is accompanied by an increase in the time required for mother cells to produce subsequent daughter cells, ultimately leading to the cessation of daughter cell production and the demise of the senescent mother cell [7]. Identification of genes regulating or at least influencing the aging process of *S. cerevisiae* is facilitated by the fact that it is characterized by a streamlined genome of approximately 12.07 Mb distributed across 16 linear chromosomes [8, 9]. Within this genome, a set of over 6000 genes has been identified, of which only around 5% harbor introns, amounting to 296 introns from 287 genes—a notably sparse distribution of introns throughout the yeast's genetic landscape [10], while a prior report suggested that *S. cerevisiae* contained only 253 introns with a mere six genes featuring two introns [11].

The influence of mRNA levels on protein expression is well-recognized, encompassing not only gene expression but also RNA decay rates [12]. RNA decay, often impacted by premature translation termination [13], becomes a pivotal aspect in the fate of messenger RNA molecules. Of particular note is alternative splicing (AS)—an intricate process yielding either novel proteins or mRNA transcripts prone to degradation via the nonsense-mediated mRNA decay (NMD) pathway. In the context of yeast, the influence of AS has been discerned since 1990, with different mRNA sequences orchestrating this process [14].

The *SUS1* gene, remarkably bearing two introns, offers an intriguing link between AS and its functional outcomes. This gene, pivotal in mRNA transport and histone H2B deubiquitination, provides an exemplar of how AS can impact gene expression and subsequent cellular processes [15]. Although the prevalence of AS in fungi is generally overshadowed by metazoan proteome diversification mechanisms, sporadic instances such as the sub-functionalization of the *SKI7/HBS1* gene demonstrate its relevance [16].

Recent research has unveiled a nuanced perspective on AS in *S. cerevisiae*. While frequent, AS in yeast is often modulated by RNA degradation, predominantly serving to regulate transcript abundance rather than expanding the proteome [17]. The *SUS1* gene again emerges as a case in point, where the non-canonical sequences within its first intron led to retention and subsequent degradation—a regulatory strategy that underscores the intricacies of splicing [15].

Intriguingly, the prevalence of introns in Ascomycetes, including *S. cerevisiae*, has been implicated in homologous recombination with reverse-transcribed RNA containing introns, reflecting the dynamic interplay between introns and genome stability [18]. Ubiquitin-like proteins, exemplified by Hub1p, contribute to AS modulation, interacting with spliceosomes and influencing their activity [19].

While instances of protein diversity resulting from AS have been documented in Ascomycetes, the intricate regulatory mechanisms underlying these events are still being unraveled. *YRA1*, a gene involved in mRNA export, utilizes intron retention (IR) as a mechanism for autoregulation, shedding light on the multifaceted roles of AS in cellular control [20]. Similarly, the *PTC7* gene undergoes AS, generating distinct protein isoforms with disparate subcellular locations—a process with implications for cellular function and compartmentalization [21].

Beyond *S. cerevisiae*, other yeast species such as *Yarrowia lipolytica* and *Schizosaccharomyces pombe* have exhibited AS capabilities, further emphasizing the evolutionary significance of this process [22–24]. The prevalence of IR has been identified as a hallmark of genome-wide age-related experiments, extending to organisms like *Caenorhabditis elegans*, where IR pertains to metabolic processes including carbohydrate transport and lipid catabolism, even under dietary restrictions [25–27].

In this study, we delve into the intricate landscape of AS in the context of yeast aging, particularly focusing on the model organism *S. cerevisiae*. By exploring the complexities of IR and functional implications, we aim to increase our understanding of AS during the aging process of yeast.

## RESULTS

To investigate the occurrence of IR during replicative yeast aging, we analyzed public poly-A RNA-seq data derived from yeast mother cells isolated at different replicative ages: 0 h, 7.8 h, 17.8 h, 45.4 h and 72.3 h (collectively referred to as *Janssens data set* [28] hereinafter). To generate this data set, Janssens *et al.* used a column system in which yeast mother cells labeled with iron beads were retained using a magnet, while the generated daughter cells were continuously washed away. Specifically, we used the data labeled as Mix 2 (ArrayExpress accession: E-MTAB-3605) as it corresponds to the column fraction enriched in aged yeast mother cells. As a quality control measure, we confirmed that the gene expression values obtained through our edgeR analysis closely matched those initially reported by Janssens *et al.* [28] (Fig. S1A). We further verified that the Janssens data set evinced transcriptional changes associated with replicative aging. Gene set enrichment analysis (GSEA) indicated enrichment of a transcriptional signature that has been identified in wild type and mutant yeast models of replicative aging [29] (Fig. S1B). Thus, we confirmed that we analyzed representative data of the transcriptional program of replicative yeast aging.

**Figure 1:**
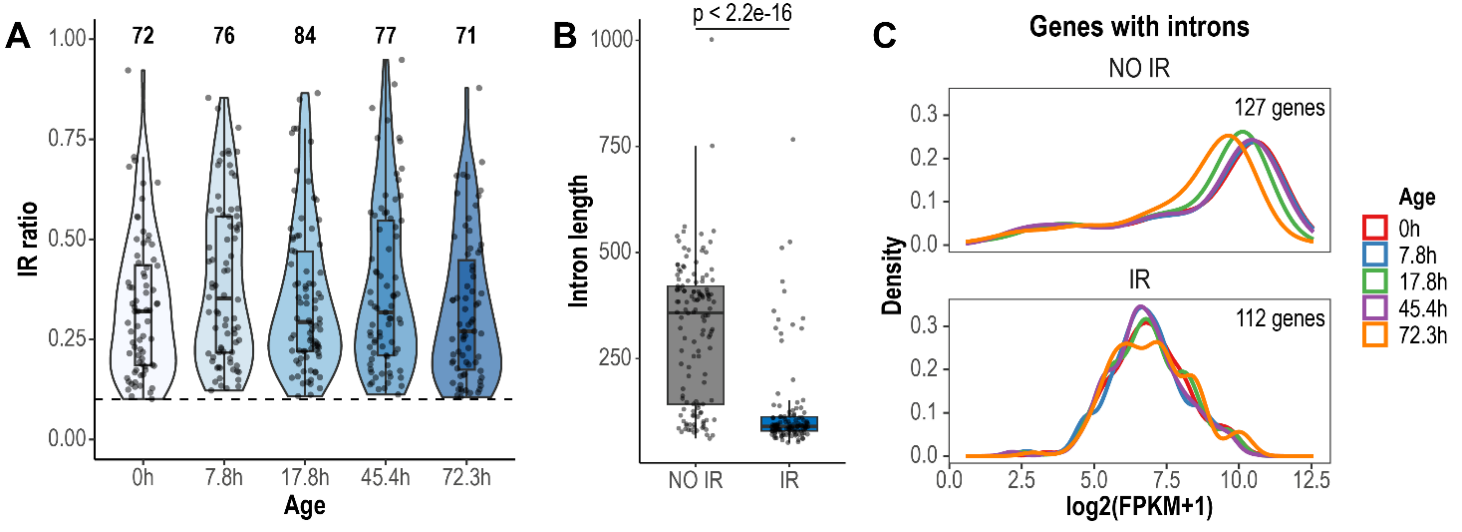
FIGURE 1: Global detection and characteristics of retained introns during replicative yeast aging. **(A)** Violin plots displaying IR ratio distribution of the 116 retained introns identified across all aging time points. Each point is an intron. The dashed line indicates the threshold value of IR ratio for an intron to be considered as retained (IR ratio > 0.1). The number of retained introns identified in each time point is indicated at the top. **(B)** Box plots displaying the distribution of the lengths of non-retained (NO IR) and retained introns (IR). Only the introns of genes with Fragments Per Kilobase of transcript per Million reads mapped (FPKM) > 1 are shown. Significance was tested using Wilcoxon rank sum test (p < 2.2e-16). **(C)** Expression levels of genes with non-retained introns and genes expressing IR transcripts. Only genes with FPKM > 1 are shown. Note that the density curves, which represent the distribution of the data, are shifted towards lower expression values in genes expressing IR transcripts.

We conducted a global analysis of retained introns using IRFinder [30]. IRFinder uses the IR ratio metric, which represents the proportion of transcripts retaining introns for a given gene. As reported in other publications [30, 31], we considered an intron as retained if it was estimated to be present in at least 10% of the transcripts (IR ratio > 0.1). In total, 116 retained introns in 112 genes were identified across all aging time points (**Fig. 1A**). Most retained introns displayed IR ratio values between 0.1-0.5 (**Fig. 1A**). Previous reports have highlighted that retained introns are shorter in length and have higher GC content, compared to non-retained introns [31, 32]. We found that introns identified as retained in the Janssens data set were significantly shorter than non-retained introns (retained introns average length = 127 bp, non-retained introns average length = 314 bp, p < 2.2e-16, Wilcoxon rank sum test; **Fig. 1B**). However, GC content was not different between retained and non-retained introns (retained introns average GC content = 32.5%, non-retained introns average GC content = 33%, p = 0.278, Wilcoxon rank sum test). Genes expressing IR transcripts displayed lower transcript levels than genes that did not retain introns (**Fig. 1C**). Of note, this previously unexplored feature of IR in yeast goes in line with previous observations in human cells [33].

**Figure 2:**
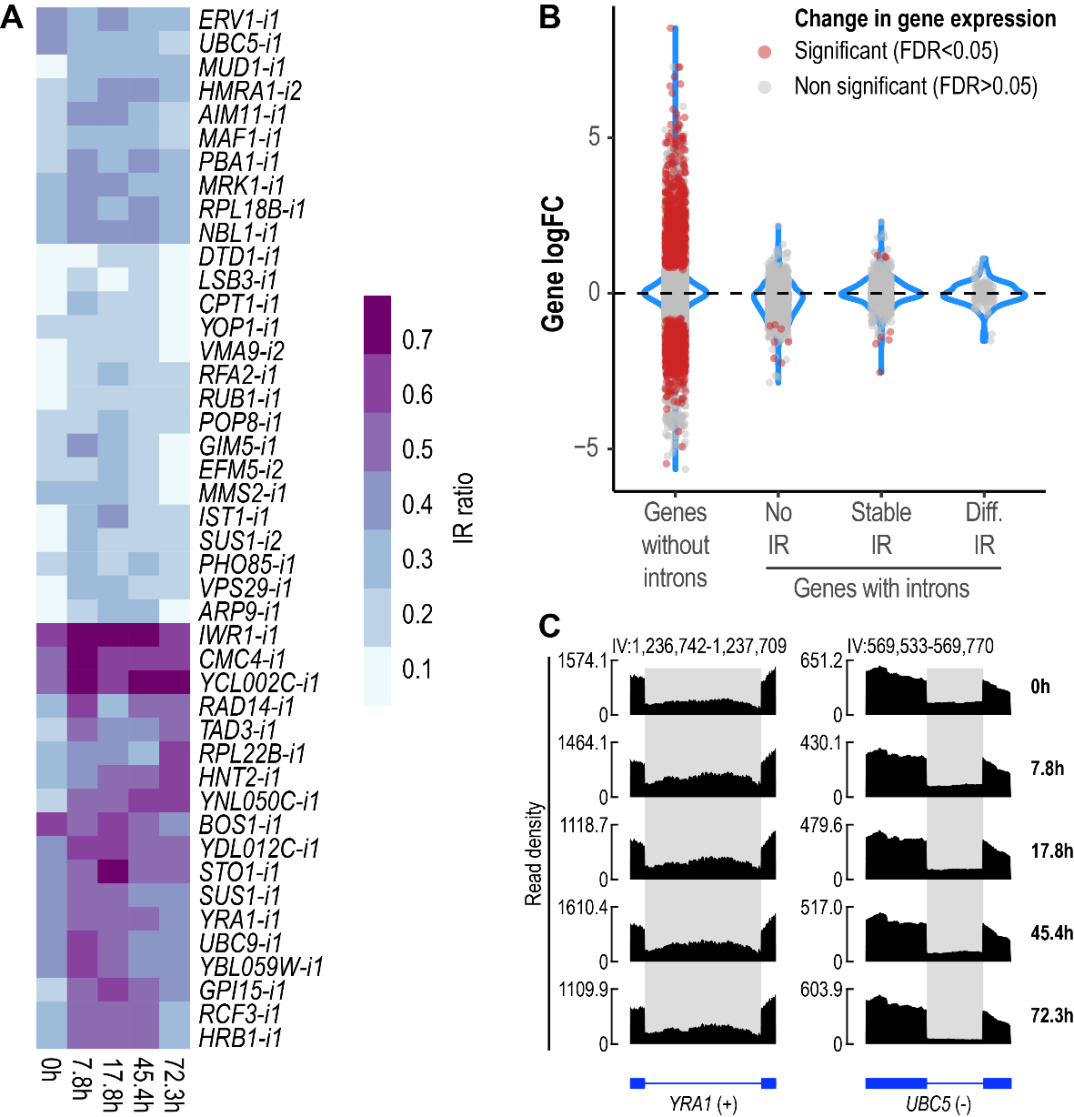
FIGURE 2: IR is altered during replicative yeast aging. **(A)** Heatmap displaying changes in IR levels of the 44 differentially retained introns identified during replicative yeast aging. **(B)** Distribution of the Log2 fold change values of genes without introns (single-exon genes) and genes with introns that show either no IR, stable IR levels or differential IR. Significant genes (False Discovery Rate (FDR) < 0.05) are shown in red. The plot includes the data of all the comparisons between the time points analyzed in this study. **(C)** RNA-seq tracks displaying changes in IR levels for *YRA1* and *UBC5*. The y-axis shows read density. In the bottom part, the annotation of each gene is shown; exons are represented as boxes and introns as lines. Differentially retained introns are highlighted in grey. A plus (+) or a minus (-) sign next to the gene name indicates that the gene is encoded by the forward or reverse strand, respectively. RNA-seq tracks were generated with SparK [76].

We then sought to identify introns with altered retention levels during replicative aging. IR was found to be a dynamic event during replicative aging. Overall, 44 differentially retained introns in 43 genes were identified across all aging time points (**Fig. 2A**, Supplemental Table 1). To gain further insight into the relevance of IR as part of the transcriptional program of replicative aging in yeast, we assessed the occurrence of other AS events using rMATS [34]. We analyzed exon skipping and alternative 5′ and 3′ splice sites, all of which are known to occur in *S. cerevisiae* [35, 36]. Apart from IR, we only found alternative 3′ splice sites to be altered (A3SS), albeit at a lower proportion (A3SS = 14 events in ten genes) (Fig. S2). These results indicate that IR represents the predominant type of AS during replicative aging in yeast.

Changes in IR levels can affect gene expression [37]. To further explore whether genes with differentially retained introns displayed altered transcript levels, we divided yeast genes into four categories (**Fig. 2B**): genes without introns (or single-exon genes), genes with non-retained introns (No IR), genes showing stable IR levels (Stable IR), and genes with differentially retained introns (Diff. IR). Notably, it was only the latter group that did not display any significant changes in transcript levels during yeast aging (**Fig. 2B**). This observation indicates that during replicative aging some genes may be functionally altered via IR without displaying changes in gene expression.

We identified genes showing differential IR that have been previously implicated in replicative yeast aging, e.g., *LSB3*, *PHO85* and *HRB1*, whose corresponding mutants display altered longevity [38]. However, we detected various other genes that have not been formally associated with aging in yeast, two of which are exemplified in **Fig. 2C**. *YRA1*, which encodes an RNA-binding protein involved in the nuclear export of mRNAs, displayed increased IR levels during the early and middle stages of aging (**Fig. 2C**, left). Conversely, *UBC5*, which encodes a ubiquitin-conjugating enzyme, showed decreased IR as age progressed (**Fig. 2C**, right). The RNA-seq tracks of all the other differentially retained introns are shown in Fig S3.

**Figure 3:**
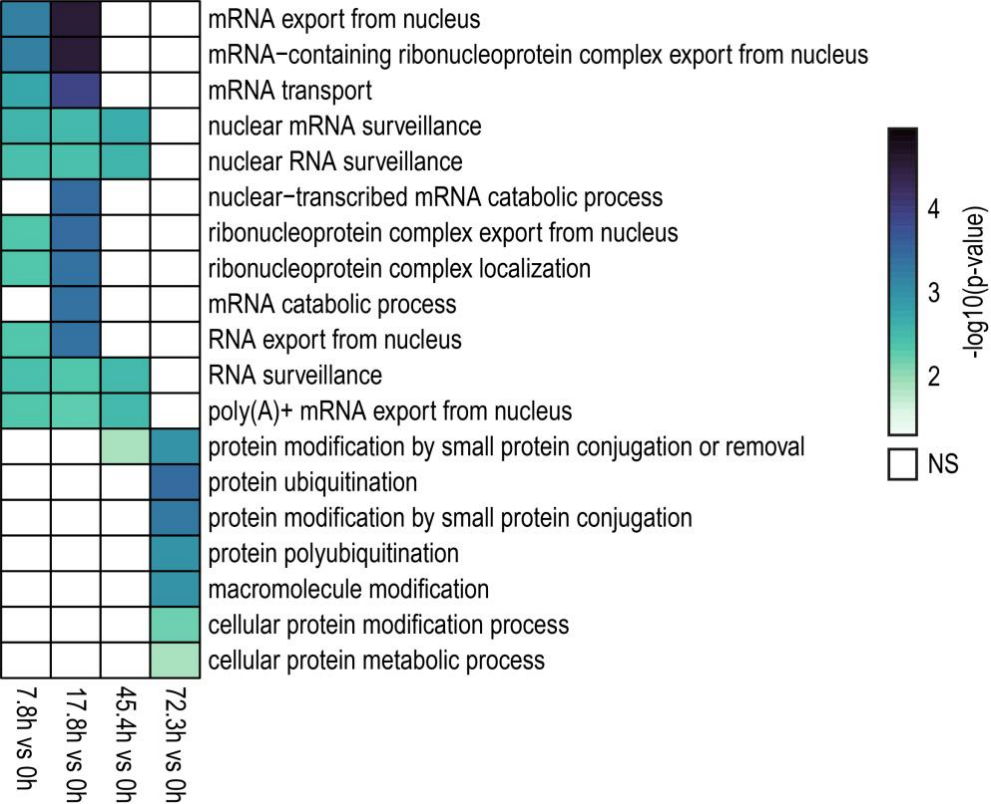
FIGURE 3: GO (Biological Process) analysis of genes showing differential IR levels during replicative yeast aging. Heatmap displaying the enriched biological processes identified for genes with differentially retained introns in the contrasts indicated in the bottom part (7.8 h vs 0 h, 17.8 h vs 0 h, 45.4 h vs 0 h, 72.3 h vs 0 h). Colored and blank boxes within the heatmap indicate significant and non-significant (NS) terms. The color scale represents the significance (Fisher's Exact test) of the term expressed as -log10 (p-value). GO terms with p < 0.05 were considered as significant. Note that during the early and middle stages of the replicative lifespan of yeast (7.8 h, 17.8 h and 45.4 h), genes with differentially retained introns were primarily involved in mRNA processing and export. On the other hand, genes exhibiting altered IR levels during the latest aging time point analyzed here (72.3 h) were mainly associated with protein ubiquitination.

A gene ontology (GO) analysis was conducted to identify enriched biological processes among genes showing differential IR levels during yeast aging (**Fig. 3**). The GO analysis revealed that during the early stages of yeast aging, genes showing differential IR were primarily associated with mRNA processing and export. On the other hand, increased yeast age was associated with altered IR levels in genes related to protein ubiquitination. Altogether, the GO analysis suggests that these processes may be functionally altered by IR during yeast aging. In fact, this may go in line with previous observations in which protein overabundance and uncoupled transcript and protein levels were observed during replicative yeast aging [28].

Our results highlight the occurrence of dynamically altered IR events during replicative aging in yeast. To further expand our findings, we analyzed two additional poly-A RNA-seq data sets of aged *S. cerevisiae* mother cells reported by Paukštytė *et al.* [39] (replicative age: 6 h) and Hendrickson *et al.* [29] (replicative ages: 20 h, 40 h). To generate these data sets, the authors labeled yeast mother cells with iron beads and recovered them using a magnet at different replicative ages, in a similar way to the approach used by Janssens *et al.* [28]. We identified 21 and 17 differentially retained introns in the Paukštytė (Fig. S4A) and Hendrickson (Fig. S4B) data sets, respectively, various of which were shared with the Janssens data set (Fig. S4C). Most differential IR events (75%) shared with the Janssens data set displayed the same pattern of increased retention during aging. Interestingly, we found overlap of differential IR events in genes associated with mRNA processing and export (e.g., *YRA1* and *STO1*) and protein ubiquitination and the proteasome (e.g., *UBC9* and *PBA1*). These results reinforce the fact that IR is an altered component of the transcriptional program of replicative yeast aging. The data of all the differentially retained introns identified in this study can be found in Supplemental Table 1.

## DISCUSSION

In this study, our goal was to analyze yeast transcriptome data for the occurrence of IR during replicative aging. Towards this end, we primarily utilized the high-quality RNA-seq data set previously reported by Janssens *et al.* [28]. For generating this data set, the authors effectively isolated aged yeast mother cells using a then novel column system and profiled their transcriptome at different time points. With this data set, Janssens *et al.* originally reported a valuable analysis of gene expression changes during replicative yeast aging. We expanded on the data previously presented by Janssens *et al.* by exploring whether old cells are marked by a different occurrence of IR compared to young cells.

IR has been relatively poorly studied in the context of aging. Only a few studies highlight the importance of IR as part of the transcriptional landscape of aging and age-related diseases [32, 40–43]. For instance, analysis of *Drosophila* heads, and human and mouse brains revealed increased IR in aged organisms [32]. Increased IR is also observed in the frontal cortex and cerebellum of Alzheimer's disease patients [32], as well as in a large number of cancer types [44]. More recently, IR has been proposed as a pre-symptomatic marker of aging [40].

In the present study, we found that IR is dynamically altered in yeast during replicative aging (**Fig. 2A**). Notably, genes with differentially retained introns did not display altered transcript levels (**Fig. 2B**). In various studies, it has been frequently observed that changes in IR levels are inversely related to changes in gene expression, e.g., increased IR results in decreased gene expression and vice versa [42, 45–47]. However, this was not the case in aged *Drosophila* heads, and aged human and mouse brains, in which genes with altered IR did not display changes in gene expression [32], which is in line with our findings for yeast aging. Based on these observations, we underscore the fact that genes with altered IR, but unaltered transcript levels, may be overlooked when conducting conventional transcriptomic analyses in aging studies. Consequently, genes potentially contributing to the onset and development of aging via IR could be potentially ignored.

An important aspect of replicative aging in yeast is the increased time mother cells take to generate daughter cells. This is ultimately accompanied by a decline in the replicative capacity of aged cells [48]. In other organisms it has been observed that, while cells that can transition into a proliferative state display lower IR levels, cells transitioning towards post-mitotic states show increased IR [49]. In the present study, of the 44 differentially retained introns that we identified in the Janssens data set, 32 displayed increased IR levels during the time course of aging (**Fig. 2A**, Supplemental Table 1). Increased IR during replicative yeast aging may be a consequence of the ongoing transition towards the cell state of reduced replicative capacity. Nonetheless, further research is required to unveil the underlying mechanisms driving increased IR during yeast aging.

We found that genes showing differential IR during replicative aging in yeast were primarily associated with the processes of mRNA processing and export, and protein ubiquitination (**Fig. 3**). Interestingly, genes with altered IR in the aged mouse brain and in the liver of a mouse model of premature aging were involved in similar processes [32, 40, 41]. These observations underscore the involvement of IR in these cellular functions as part of the transcriptional program of aging across species.

Various mRNA export genes that we identified, such as *SUS1*, *STO1*, *YRA1* and *HRB1* displayed increased IR during yeast aging (**Fig. 2A**). The Yra1p and Hrb1p proteins work conjointly during transcription and mRNA export [50]. IR in *YRA1* transcripts introduces a premature termination codon that triggers their degradation as a mechanism to regulate Yra1p protein levels [20]. Yra1p is a key mRNA export factor in yeast as *YRA1* mutants display marked export defects [51, 52]. In contrast, Δ*hrb1* mutants do not show impaired mRNA export, but rather aberrant leakage of IR transcripts into the cytoplasm [53]. This underscores the role of Hrb1p in nuclear RNA surveillance and mRNA export quality control. Notably, *HRB1* has been previously associated with aging in *S. cerevisiae* as Δ*hrb1* mutants display increased longevity [38]. Several studies in other species, such as human and mouse, have highlighted the occurrence of IR in RNA-binding protein genes involved in various functions, such as splicing, mRNA export, and polyadenylation of mRNA [44, 54–58]. For instance, in human motor neurons carrying mutations that cause amyotrophic lateral sclerosis, a disease largely dependent on age, the most prominent IR event was observed in the *SFPQ* gene, which is involved in transcription and splicing [57].

On the other hand, we found that protein ubiquitination genes, such as *UBC5* and *MMS2* displayed decreased IR during the latest aging time point analyzed here (**Fig. 2A**). In contrast, IR levels in *UBC9* transcripts peaked during early stages of aging and returned to “basal” levels at later time points. In addition, *PBA1* transcripts, which encode a chaperone involved in the assembly of the 20S proteasome, also displayed increased IR during aging. The ubiquitin-proteasome pathway (UPP) is known to be impaired during aging [59]. In fact, loss of proteostasis is a recognized hallmark of aging and age-related diseases, such as Alzheimer's and Parkinson's disease [60]. In yeast, impaired proteasome activity and increased accumulation of ubiquitinated proteins are observed during aging [61]. Moreover, yeast with enhanced proteasome function display increased longevity [62]. Although *UBC5* has not been formally associated with yeast aging, it has been proposed as a potential pro-longevity gene using predictive models based on functional annotation and gene expression data [63].

Previously, Janssens *et al.* [28] reported an intriguing finding during replicative yeast aging in that they observed that as age increases there is less agreement between transcript and protein levels, meaning that transcriptional changes did not correlate with alterations observed in the proteome. We suggest that altered mRNA export and protein degradation as a result of the dynamic IR patterns identified here may contribute to the discrepancies between transcript and protein levels previously observed in replicative aging in yeast [28]. Although it has not yet been studied whether altered IR in mRNA export genes impairs the transport of transcripts into the cytoplasm, previous observations in *S. cerevisiae* have highlighted that deletion of the *YRA1* intron results in nuclear accumulation of mRNAs and slowed growth [64]. Therefore, dysregulation of IR events in genes here identified such as *YRA1*, *HRB1* or *SUS1* could have profound consequences in the availability of cytoplasmic transcripts ready for translation, thus potentially affecting the proteome of aged yeast.

By comparing various data sets of replicative aged yeast, we found increased IR in *GPI15* transcripts as a shared event in all data sets (Fig. S4C). *GPI15* encodes a protein involved in the biosynthesis of glycosylphosphatidylinositol anchors, which are post-translational modifications added to some membrane proteins [65]. While there is no reported association between *GPI15* and yeast aging, a functionally related gene *GPI7* has been predicted to impact yeast lifespan [66]. Furthermore, in another study in which the unfolded protein response pathway was compromised in yeast, the expression of *GPI15,* and other functionally related genes, was found to be altered [67]. It was hypothesized that changes in the expression of these genes may aid in counteracting endoplasmic reticulum stress [67]. Notably, the unfolded protein response, and consequently proteostasis, are impaired during aging, resulting in increased endoplasmic reticulum stress [68, 69], in which IR in *GPI15* transcripts may play a role as yeast age progresses. Further research into the occurrence of IR in *GPI15* transcripts during yeast aging is required to better understand the functional consequences of this widespread event.

One limitation of our study is the fact that we utilized the raw, “mixed” RNA-seq data reported by Janssens *et al.* [28]. Originally, the authors conducted an “unmixing” step, in which they removed the potential influence of the transcriptome of daughter or dead cells present in the samples that were enriched for aged yeast mother cells. However, during the “unmixing” step the intron information was lost as the samples were unmixed at the gene expression level. For our study, we required the information of introns which was still available in the raw data set. Nonetheless, we found that IR is altered in other studies of replicative yeast aging and some of these differential IR events were shared with the Janssens data set (Fig. S4), further supporting our observations that IR is part of the transcriptional program of replicative aging in yeast.

Our work highlights the role of changes in transcriptional regulation during aging. This might also hold true for other organisms besides yeast. As outlined above, it has been shown that IR is increased in *Drosophila* heads, and human and mouse brains during aging [32]. Since yeast is characterized by chronological aging and replicative aging, it would be straightforward to analyze IR during stationary phase survival, which only depends on the availability of a high-quality transcriptome data set. We assume that several important regulatory principles can be identified by our approach, which ultimately might even guide interventions into the aging process.

## MATERIALS AND METHODS

### Data collection and processing

RNA-seq data from replicative aged yeast was retrieved from the study by Janssens *et al.* [28] (strain: YSBN6; ArrayExpress accession: E-MTAB-3605). This dataset was selected since it met various criteria for the proper analysis of AS and IR in yeast aging: (1) enrichment and isolation of aged yeast mother cells was effectively conducted, (2) poly-A RNA was enriched during library preparation, and (3) sequencing was paired-end. Five aging time points with two replicates each were analyzed: 0 h, 7.8 h, 17.8 h, 45.4 h and 72.3 h. Two additional RNA-seq data sets of wild type replicative aged yeast that met the above-mentioned criteria were obtained from the studies by Paukštytė *et al.* [39] (strain: BY4741; replicative age: 6h; Harvard Dataverse: https://doi.org/10.7910/DVN/DUOBUD) and Hendrickson *et al.* [29] (strain: DBY12000; replicative ages: 20h and 40h; GEO accession: GSE118581). Read quality was assessed with FastQC v0.12.1. Low quality bases (quality score < 30) and sequencing adapters were trimmed using Cutadapt v4.4. STAR v2.7.10b [70] was used to map the filtered reads against the R64 *S. cerevisiae* genome. Since the size of the yeast genome is 12.1 Mb, the parameter *--genomeSAindexNbases* was set to 10 when indexing the genome.

### Differential gene expression analysis

Gene counts were generated using featureCounts (Rsubread package v2.14.2) together with the *S. cerevisiae* ENSEMBL gene annotation (release 109). Normalization of gene counts, computation of FPKM values, and differential gene expression analysis were conducted with edgeR v3.42.4 [71]. Genes showing FDR < 0.05 were considered as differentially expressed.

### Correlation analysis

To make sure that the gene expression data here generated using our pipeline was consistent with the original raw analysis conducted by Janssens et al. [28], a correlation analysis was conducted. Log2 fold change values for all contrasts against 0h were computed using the raw FPKM values reported in Janssens *et al.* [28] (source data 2); the same was performed with the FPKM values here generated. Spearman correlation analysis was conducted for genes identified as differentially expressed in our pipeline (7.8 h vs 0 h, 122 genes; 17.8 h vs 0 h, 186 genes; 45.4 h vs 0 h, 301 genes; 72.3 h vs 0 h, 546 genes).

### Gene set enrichment analysis of aging genes

Custom yeast aging gene sets were generated using the top 100 up- and downregulated genes reported in the analysis by Hendrickson *et al.* [29], in which DBY12000 wild type (WT) and three mutant (Δ*sir2*, Δ*ubr2*, Δ*fob1*) yeast strains were profiled during replicative aging. edgeR normalized expression values for all genes here analyzed were introduced to the GSEA tool v4.2.2 [72]. GSEA was run by setting the *Metric for ranking genes* parameter to log2_Ratio_of_Classes as suggested when analyzing experiments with less than 3 replicates. Significant gene sets were considered as those with p-value < 0.05 and FDR < 0.25.

### Intron retention analysis

IRFinder v1.3.1 [30] was used for the analysis of retained introns. IRFinder uses the IR ratio metric to estimate IR levels and is equivalent to the proportion of transcripts retaining introns. IRFinder reference was generated using the *S. cerevisiae* ENSEMBL gene annotation (release 109). IR was assessed in 253 introns of 243 genes. In the case of the Janssens data set (two replicates per time point), differential IR analysis was conducted using the *analysisWithLowReplicates.pl* script (Audic and Claverie test [73]). For the Paukštytė (four replicates per time point) and Hendrickson data sets (three replicates per time point), differential IR analysis was conducted using the Generalized Linear Model approach with DESeq2 [74] (Likelihood Ratio Test). Differentially retained introns were considered as those showing |ΔIR ratio| > 0.1 and p-value < 0.05.

### Alternative splicing analysis

rMATS v4.1.2 [34] was used to identify AS events other than IR, e.g., exon skipping and alternative 5′ and 3′ splice sites. rMATS was run using the --*novelSS* parameter. The output was further filtered using maser v1.18.0 to keep AS events with at least five reads supporting their expression, and a modified version of the *topEvents()* function was used to identify differential AS events as those showing |ΔPSI| > 0.1 and p-value < 0.05 (PSI, Percent Spliced In).

### Gene ontology analysis of genes with differential IR

Gene ontology (GO) analysis was conducted for genes showing differentially retained introns using DAVID (https://david.ncifcrf.gov/) [75]. Significantly enriched biological processes (p < 0.05, Fisher's Exact test) were identified using the GOTERM_BP_ALL category.

## AUTHOR CONTRIBUTION

A.d.O.F.d.V. conceived the study. J.G.M. performed most analyses. L.F.D.l.C.G. conducted the GO analysis. J.G.M., A.d.O.F.d.V. and C.Q.S. prepared the first draft of the manuscript with contributions from L.F.D.l.C.G. A.d.O.F.d.V. and C.Q.S. supervised the work. J.M.G.M. supervised the work and provided intellectual and strategic input to the analyses and manuscript. All authors have read and approved the final manuscript.

## SUPPLEMENTAL MATERIAL

Click here for supplemental data file.

Click here for supplemental data file.

All supplemental data for this article are available online at www.microbialcell.com/researcharticles/2024a-gomez-moltavo-microbial-cell/.

## Abbreviations:

A3SS: – alternative 3′ splice sites,
AS: – alternative splicing,
GO: – gene ontology,
IR: – intron retention.
