# Supplementary material for: Replicative aging in yeast involves dynamic intron retention patterns associated with mRNA processing/export and protein ubiquitination
Source: Microb Cell. 2024 Feb 23;11:69–78. doi: 10.15698/mic2024.02.816 (PMC10897858; doi:10.15698/mic2024.02.816)
Supplement: Supplementary file 2 [file mic-11-069-s02.pdf]

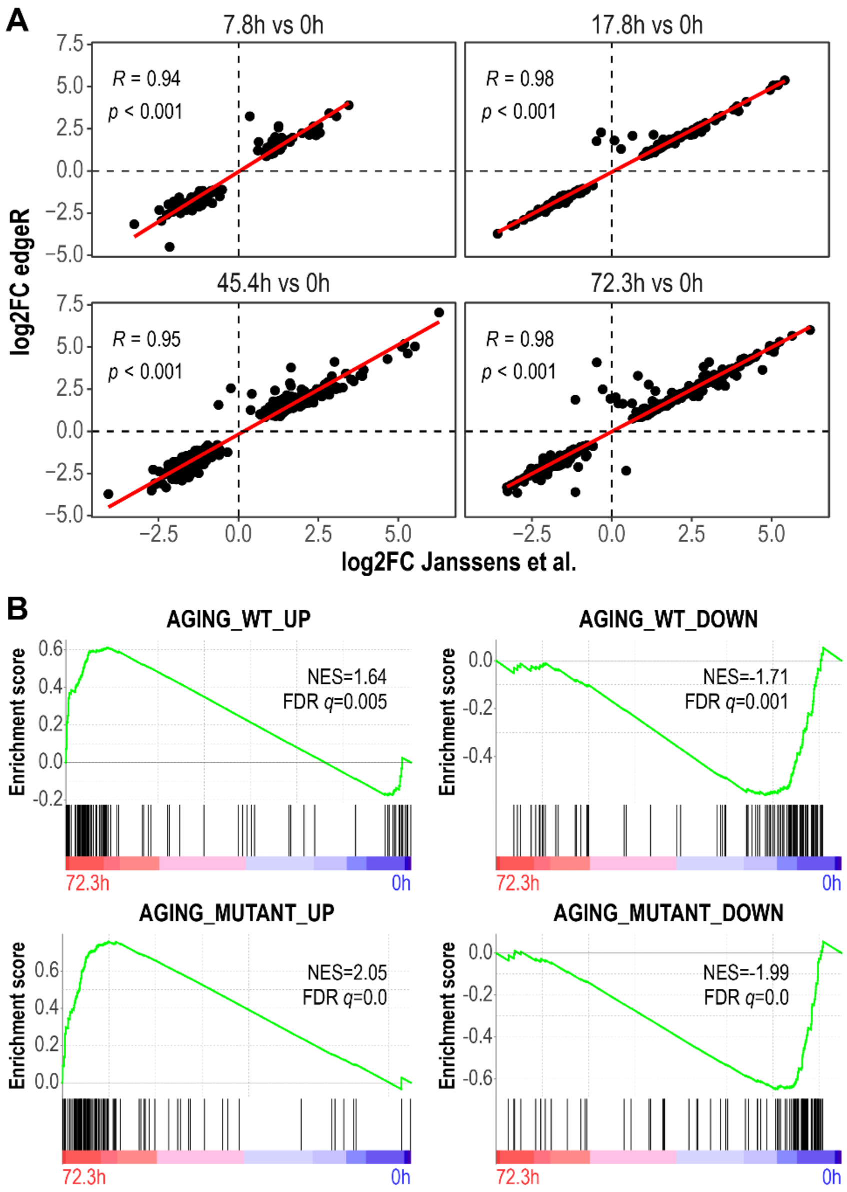

FIGURE S1: Quality control on Janssens data prior to the analysis of IR events. (A) Spearman correlation between log2 fold change values obtained by our processing pipeline and the original analysis by Janssens et al. [28]. The analysis was conducted only using the genes identified as differentially expressed in our analysis (*see Methods*). **(B)** GSEA showing that the data here used showed an aging-associated transcriptional signature identified in wild type and mutant models of replicative yeast aging in Hendrickson et al. [29]. The y-axis displays the enrichment score. The color bar in the bottom part of each plot represents genes ranked from the most upregulated (red) to the most downregulated (blue) genes at 72h compared against 0h. Each vertical bar above the color bar represents a hit of the customized aging gene set (*see Methods*). Note that for the AGING_WT_UP and AGING_MUTANT_UP gene sets, the enrichment curve (green) is biased towards the red (left) portion of the color bar which indicates that upregulated genes in the Janssens data set were enriched in genes that have been identified with increased expression in other models of replicative yeast aging [29]. NES, Normalized Enrichment Score.

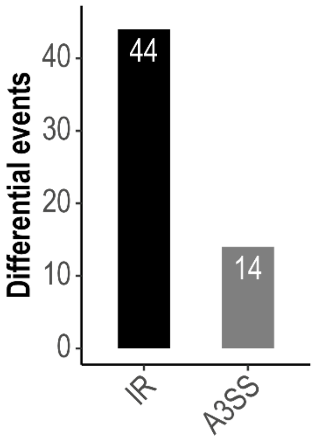

FIGURE S2: IR is the predominant type of AS during replicative yeast aging. Bar plot showing the number of differential IR and A3SS events identified across all aging time points. IR = 44 differentially retained introns in 43 genes. A3SS = 14 differential events in ten genes.

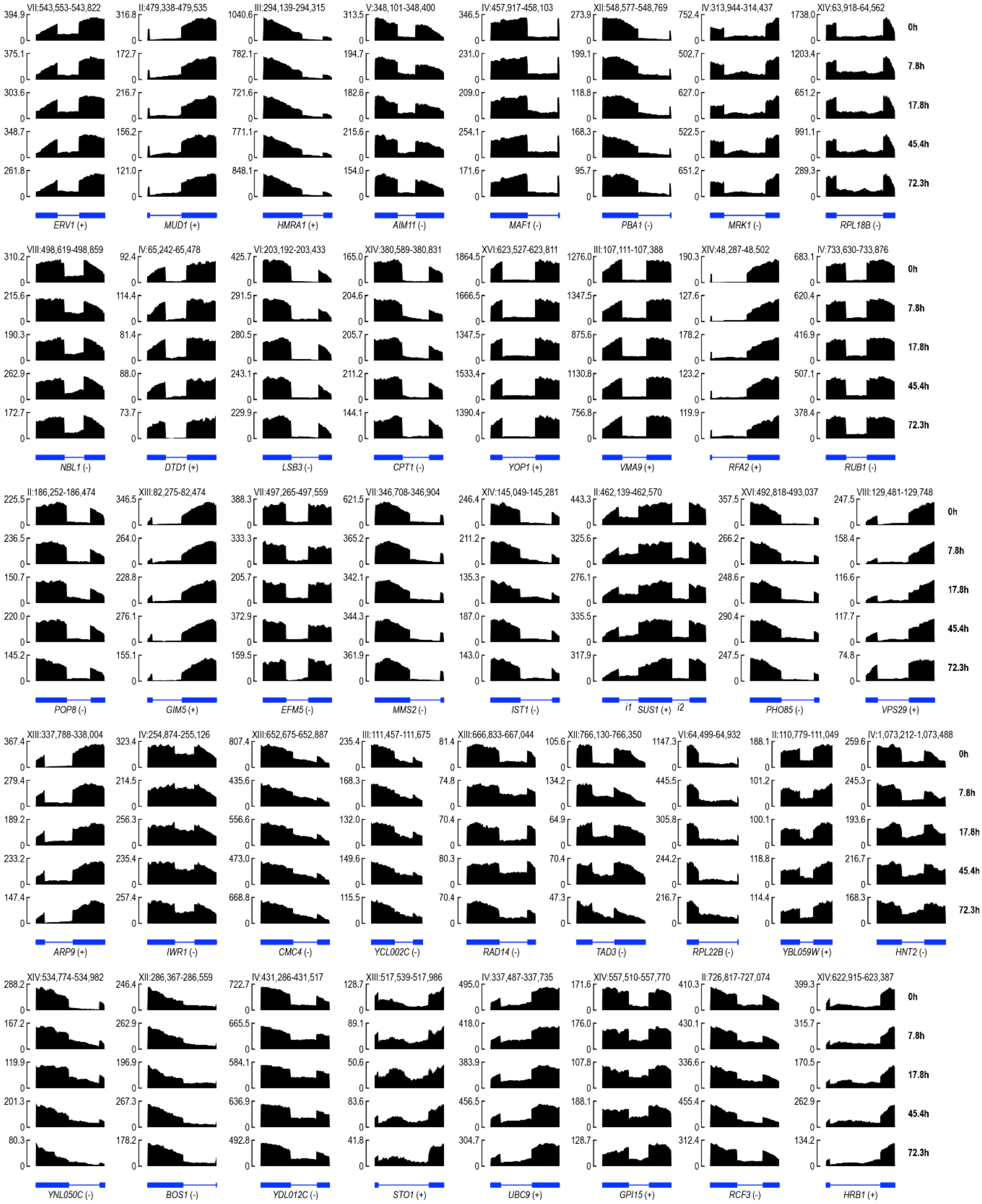

FIGURE S3: Differentially retained introns identified during replicative yeast aging. The panel shows the RNA-seq tracks of the differentially retained introns presented in Fig. 2A for the Janssens data set. In the case of the *SUS1* gene (third row), both introns are shown. The y-axis shows read density. In the bottom part, the annotation of each gene is shown; exons are represented as boxes and introns as lines. A plus (+) or a minus (-) sign next to the gene name indicates that the gene is encoded by the forward or reverse strand, respectively. RNA-seq tracks were generated with SparK [76].

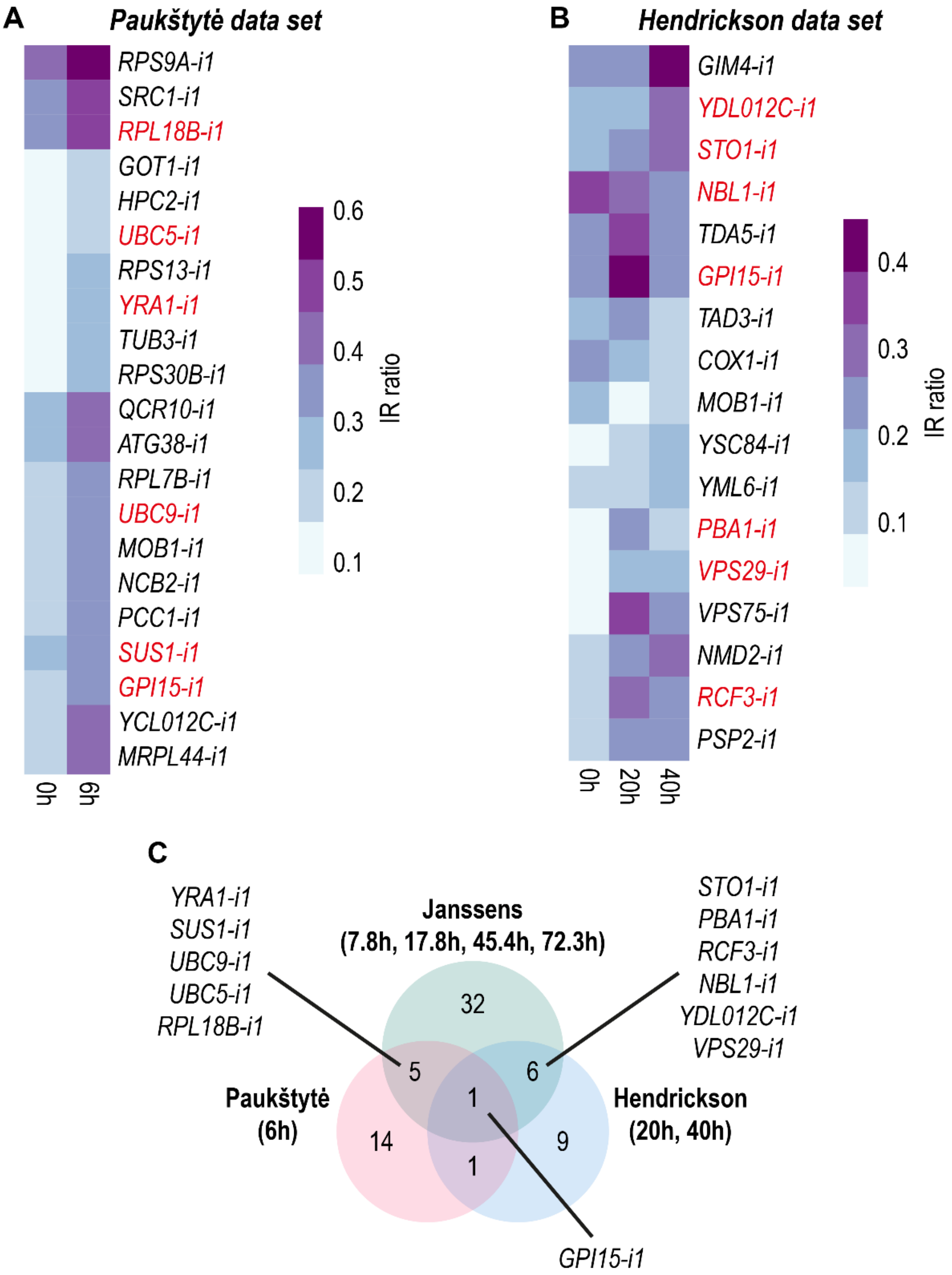

FIGURE S4: Differentially retained introns are identified in other studies of replicative yeast aging. Heatmaps displaying changes in IR levels of the differentially retained introns identified in the RNA-seq data sets published by **(A)** Paukštytė et al. [39] and **(B)** Hendrickson et al. [29]. Differential IR events shared with the Janssens data set are highlighted in red. **(C)** Venn diagram showing the number of differential IR events shared between the Janssens, Paukštytė and Hendrickson data sets.
